# Nurses’ usage of validated tools to assess for delirium in general acute care settings: A scoping review protocol

**DOI:** 10.12688/hrbopenres.14081.1

**Published:** 2025-02-13

**Authors:** Aoibhinn Boyd, Marcia Kirwan, Leona Bannon

**Affiliations:** 1 Dublin City University School of Nursing Psychotherapy and Community Health, Dublin, Leinster, Ireland

**Keywords:** Delirium, Validated assessment tools, Nursing, Missed care, General acute care settings, Scoping review

## Abstract

**Background:**

Delirium is an acute, neuropsychiatric syndrome, characterized by an altered mental state. It often affects hospital in-patients and is associated with an increased risk of mortality, dementia, and functional decline. Delirium can be detected through the use of validated assessment tools, administered by nurses, and early detection is associated with improved outcomes for patients. However, validated tools are infrequently utilised and cases of delirium are frequently missed. A greater understanding of nurses’ use of validated delirium assessment tools is needed in order to reduce the number of missed cases.

**Objectives:**

The aim of this scoping review is to identify how validated assessment tools are used by nurses in general acute care settings to assess for delirium and identify the barriers and enablers for said tools’ use.

**Methods:**

This scoping review will be conducted in accordance with the Joanna Briggs Institute methodology for scoping reviews. The databases CINAHL, PubMed, Web of Science, and Scopus will be searched using a search strategy. Grey literature will also be searched using Google Scholar and BASE. Results will be uploaded to Covidence where the sources will be screened for relevance. Data from relevant sources will be extracted using a data extraction tool.

**Results:**

The PRISMA-ScR flow diagram will present the results of the search. Results will be mapped descriptively and presented as both tabulated results and a narrative summary.

**Conclusion:**

This protocol outlines the structure of a scoping review that will analyse the existing literature surrounding nurses use of validated delirium assessment tools. This review aims to map the evidence of delirium assessment tool utilisation by nurses and identify any barriers to usage. This will support future researchers and policy makers in the improvement of delirium assessment in acute care settings.

## Introduction

Delirium is an acute syndrome, characterised by altered levels of cognition, awareness, and attention ( *Diagnostic and statistical manual of mental disorders: DSM-5.* Fifth edition, 2013). It is a major concern for public health as it is associated with a number of adverse effects, including a twofold increased risk of mortality, and a variety of cognitive and functional impairments. These include an increased risk of dementia, memory and concentration problems, sleep disturbances, institutionalisation, and need for long-term care ( Bickel *et al.*, 2008; Davis *et al.*, 2012; Koster *et al.*, 2009; Witlox *et al.*, 2010). Additionally, there are negative impacts on the hospital macroeconomics, as delirium is associated with longer hospital stays, an increased chance of readmission and increased financial costs ( Kinchin *et al.*, 2021; Lim *et al.*, 2023). There is no one defined pathophysiology for delirium, however it is hypothesised that there are numerous pathological factors that interact with each other and it is the specific types of interactions that result in the various presentations of delirium ( Maldonado, 2018). There are, however, a number of well-established risk factors for delirium that have been identified within the literature. Increased age, dementia, and comorbid physical illnesses are known predisposing risk factors for delirium ( Lim *et al.*, 2023; MacLullich & Shenkin, 2019; Tieges *et al.*, 2021). There are also a number of precipitating risk factors; urinary catheterisation, polypharmacy, and surgery ( MacLullich & Shenkin, 2019; Tieges *et al.*, 2021). These factors often can occur in general acute hospital settings. Additionally, hospitalisation itself and prolonged hospital stays are associated with the development of delirium ( MacLullich & Shenkin, 2019).

Prevalence rates of delirium vary greatly within the literature depending on the population of the study. However, the estimated occurrence rate in hospitalised adults is 10–30% ( Bellelli *et al.*, 2014; Tieges *et al.*, 2021; Titlestad *et al.*, 2024; Waszynski *et al.*, 2024). A prospective cohort study found that 17.7% of patients aged 70 and over had delirium upon admission to the hospital and an additional 11.6% of patients aged 70 and over developed delirium during their stay at the hospital ( Dolan *et al.*, 2023). Early detection of delirium, both for patients who arrive at a general acute care setting and for those who develop it in a general acute care setting, is crucial. Patients with unrecognised delirium are at a much higher risk for mortality upon discharge than patients with recognised delirium ( Lee *et al.*, 2022) and early diagnosis is a vital step in the treatment of delirium and is necessary in order to achieve the best outcomes ( Zoremba & Coburn, 2019). However, despite the high occurrence of delirium and the importance of swift diagnosis, it is estimated that 50–75% cases are missed in such general acute care settings ( Pezzullo *et al.*, 2019).

Delirium can be detected through the use of assessment tools or instruments. There are a variety of different assessment tools that have been designed that can screen for delirium in different environments and for different purposes. Many of these aim to be rapid and require little training, with the goal of being used multiple times a day ( Liu *et al.*, 2023). Guidelines regarding what validated assessment tools to use will vary by country and care setting. Wales and England follow NICE guidelines, a comprehensive set of guidelines that document general acute care setting protocol from the admission of a patient to the treatment of delirium. They support the 4AT assessment, unless a patient is in critical care or the recovery room when the CAM-ICU is recommended ( National Institute for Health and Clinical Excellence, 2023). However, the existence of guidelines does not necessarily equate to consistent assessments. Ankravs *et al.* (2020) found that while the majority of general acute care settings in Australia and New Zealand have documented protocols, policies, or guidelines regarding the daily assessment for delirium, only just over half of patients received a delirium assessment. Nurses are a key component to delirium diagnosis in general acute care settings. As they provide around-the-clock care for the patient they are in a position to identify any fluctuations that indicate the need for a validated delirium assessment ( Brooke & Manneh, 2018). Additionally, many validated delirium assessment tools are designed to be administered by nurses ( Grover & Kate, 2012), or designed not to require specialised training ( Bellelli *et al.*, 2014). However, as previously stated, delirium cases are frequently missed in general acute care settings which can have extremely negative outcomes for the patient. One explanation for this could be the underutilisation of validated delirium assessment tools in hospital wards. Azizi *et al.* (2024) found that almost 60% of Irish wards used personal judgement as the method of assessment for delirium. The 4AT, an assessment tool that is recommended for use in Irish and British general acute care settings, has a sensitivity of 89.7% and specificity 84.1% ( Bellelli *et al.*, 2014). Validated assessment tools are far more reliable than personal judgement when assessing delirium, and this was demonstrated in Azizi *et al.* (2024) study as patients that were assessed with validated tools had a higher chance of being diagnosed with delirium than patients that were assessed with personal judgment. An increase in the usage of validated assessment tools has the potential to reduce the number of missed delirium cases seen in hospitals.

Despite delirium being recognised as a neurological condition for millennia, research on the topic is still relatively novel ( Slooter, 2017). Therefore, there is limited work done on understanding the utilisation of validated delirium assessment tools. Much of the literature that exists focuses exclusively on intensive care units, postoperative delirium, or other specific contexts. There has been little done to have a broad overview of the phenomenon and that is what this scoping review aims to address. This review will build on what is already known about nurses' use of validated assessment tools and attempt to ascertain what is being done, what is not being done, and the reasons behind both.

## Review question

This scoping review will identify how validated assessment tools are used by nurses in general acute care settings to assess for delirium, and the barriers and enablers for said tools’ use.

The questions that will contribute to the stated aim are:

1. Is there evidence of the widespread usage of validated delirium assessment tools in general acute care settings by nurses?
2. What delirium assessment tools are most commonly used by nurses in general acute care settings?
3. What are the barriers and enablers to using validated delirium assessment tools that nurses experience in general acute care settings?

### Population

Eligible studies for this scoping review will focus on registered nurses who are working in general acute hospitals ( Table 1). The aim of this scoping review is to gain a deeper understanding of nurses’ use of delirium validated assessment tools as nurses are an integral aspect of delirium prevention, diagnosis, and treatment ( Waszynski *et al.*, 2024). Hospital admission, as well as various procedures that occur in a hospital setting, are risk factors for delirium ( MacLullich & Shenkin, 2019). Therefore, only nurses working in this context will be considered.

**Table 1.** Eligibility Criteria.

	Inclusion	Exclusion
Population	Sources that report on registered nurses working in general acute hospitals	Sources that report on healthcare workers that are not registered nurses
Concept	Sources that primarily examine delirium validated assessment tools	Sources that do not primarily examine delirium validated assessment tools
Context	Sources that report on nurses who are employed in an adult ward in an general acute hospital	Sources that report on nurses in paediatric wards or in non-acute hospital settings
Types of Sources	Types of sources will be left open to include both peer-reviewed studies and grey literature	Duplications of sources already included
Language	Sources that are available in English	Sources that are not available in English
Time Period	Sources produced between 2000 and 2025	Sources produced before 2000

### Concept

The concept of interest is the use of validated assessment tools for the detection of delirium in hospitals. The delaying of a delirium diagnosis is associated with the worsening of outcomes for the patient ( Kakuma *et al.*, 2003). Therefore, regular and consistent assessments of at-risk patients by nurses are necessary. There are a variety of validated assessment tools available to assess for delirium in hospitals ( MacLullich & Shenkin, 2019). However, assessments are not made as regularly as recommended, and even more rarely with validated tools ( Azizi *et al.*, 2024). This scoping review will focus on the phenomenon of the utilisation of delirium validated assessment tools by nurses in general acute hospitals. Studies that examine any aspects of nurses’ experiences using validated tools will be considered for this scoping review. All validated and non-validated assessment tools will also be included for consideration.

### Context

The context of this scoping review is adult wards in general acute hospitals. While delirium does occur in children, there are different validated assessment methods which will not be targeted in this review ( Grover & Kate, 2012). There will be no geographical limit on the inclusion criteria.

### Types of sources

All study methodologies will be considered for this review, including quantitative, qualitative, and mixed methods. Grey literature, such as reports, and opinion papers will also be considered. This review aims to provide a comprehensive summary of all information pertaining to nurses’ use of delirium validated assessment tools, requiring the types of evidence left open.

## Methods

A scoping review was considered to be most suitable for this research for a variety of reasons. Firstly, the aim of this research is to map the existing knowledge base. Secondly, the area of interest is novel. Finally, the research question includes a broad population, concept, and context. Therefore, a scoping review is most appropriate. ( Arksey & O’Malley, 2005). This scoping review will be conducted in accordance with the Joanna Briggs Institute (JBI) methodology for scoping reviews ( Peters *et al.*, 2020). The Preferred Reporting Items for Systematic reviews Meta-Analysis extension for Scoping Reviews (PRISMA-ScR) will be used to guide the reporting of this review ( Tricco *et al.*, 2018). This review will follow the nine stages of conducting a scoping review as outlined by the JBI methodology. The first three stages have been complete; determine the objectives and questions of the research; create an inclusion criteria; and develop a strategy for the searching, selection, extraction, and presentation of the evidence. Stages four to eight will involve the evidence search, selection, extraction, analysis, and presentation. Finally, stage nine will summarise the evidence in the context of the research question, draw conclusions, and address any implications of the findings. ( Peters *et al.*, 2020). A preliminary search of CINAHL, PubMed (MEDLINE), Scopus, and Web of Science was conducted and there are no existing scoping reviews on the topic. This scoping review protocol was registered on the 15th of January 2025 on Open Science Framework. Registration DOI: https://doi.org/10.17605/OSF.IO/6QEGB

### Search strategy

An initial search was carried out on CINAHL and PubMed (MEDLINE) using keywords from the research questions. Relevant articles were identified and an analysis of the text used in the titles and abstracts supported the development of the sample search strategy. This was achieved through the support of a Dublin City University librarian ( Table 2). The librarian will continue to be consulted for the development of the final search strategy and throughout the study selection to ensure that all relevant sources have been considered ( Pawliuk *et al.*, 2021). The final search strategy will be a combination of Boolean Operators and subject headings that were also identified in the initial search. The databases that will be used for this scoping review are CINAHL, PubMed (MEDLINE), Web of Science, and Scopus. The search strategy will be tested for each database and adapted as necessary. Searches will be limited to titles and abstracts. The citation lists of all included sources will also be searched for additional sources by the research team. Grey literature will be searched using Google Scholar and BASE (Bielefeld Academic Search Engine) with an adapted search strategy. The inclusion of grey literature will ensure that the search is truly systematic and the citation lists of such sources could provide the research team with other underlying data sources ( Pawliuk *et al.*, 2021). Only sources that are available in English will be considered as it is not feasible for the researchers to translate sources. Only sources published since 2000 will be considered. Delirium research is still relatively novel, despite it being a long recognised condition ( Slooter, 2017). Prior to the 21st century, a wide and inconsistent range of terminology was used when defining, diagnosing, and recording delirium, creating large barriers to cohesive research ( Morandi *et al.*, 2008). However, since the publication of the DSM-IV-TR in 2000, there has been a significant effort among researchers to standardise the language around delirium which has subsequently allowed for the research team to decide on the stated time frame ( Morandi *et al.*, 2008).

**Table 2.** Search Strategy Table.

Search	Free Text Search Terms
S1	Delirium OR “acute confusional state” OR encephalopathy OR “cognitive disorder” OR “acute confusion” OR “disorientation”
S2	Instrument OR exam* OR scale
S3	Assess* OR screen* OR diagno* OR detect
S4	S2 AND S3
S5	4AT OR “confusion assessment” OR CAM OR AMT
S6	S4 OR S5
S7	Nurs*
S8	S1 AND S6 AND S7

### Study selection

Following the search of all databases, all identified sources will be uploaded to Covidence. Covidence is a literature review tool that is endorsed by the JBI ( Peters *et al.*, 2020). Upon uploading, all duplications will be removed. Two members of the research team will independently review all titles and abstracts against the predetermined eligibility criteria. The researchers will have the option to deem a source as potentially relevant or not potentially relevant. Sources not deemed potentially relevant will be excluded at this stage. Any disagreements between the researchers about including or excluding a source will be solved through a discussion with each other or the input of the third member of the research team. At this stage, all potentially relevant sources will have their full text retrieved. In line with JBI guidelines, a pilot test will be conducted ( Peters *et al.*, 2020). The full research team will screen the full text of 25 studies or 10% of studies, whichever is larger, against the eligibility criteria. Any discrepancies will be discussed with the entire team and the eligibility criteria will be modified if necessary. Once the agreement rate is above 75%, the screening of full texts will begin. Two members of the research team will independently review the full text of all potentially relevant sources. Once again, any disagreements between the researchers about including or excluding a source will be solved through a discussion with each other or the input of the third member of the research team. Reasons for the exclusion of sources will be recorded and reported in the scoping review. Backwards and forward citation will be conducted on all included full-text sources. These citations will undergo the same process as detailed above. The results of the search and selection process will be reported in full using the PRISMA-ScR flow diagram. 

### Data extraction

A data extraction tool will be developed by the research team to support the process of information extraction. Key information that will be extracted will be the name of the authors; year of publication; country of origin; study design; aim of the study; the population studied; the delirium assessment tools investigated; and the key findings that relate to the review’s research questions. A sample data extraction tool can be seen at Table 3. However, data extraction is an iterative process and as extraction occurs the tool may be updated to reflect the information found in the studies. To ensure the tool is effective, it will be trialled by two members of the research team on 10% of the sources. Any disagreements will be solved through discussion or the input of the wider research team. Any adaptations to the tool will be reported in the scoping review.

**Table 3.** Sample Data Extraction Tool.

Author
Year of Publication
Country of Origin
Study Design
Aim of Study
Nurse Population
Patient Population
Delirium Assessment Tool(s) Investigated
Type of Ward(s)
Key Findings

### Data analysis and presentation

To answer the questions of this scoping review, and in line with the aim of scoping reviews, the results will be mapped descriptively. The primary researcher will analyse the data extracted and will present the results as both tabulated results and a narrative summary. The PRISMA-ScR flow diagram will present the results of the search. There will be a descriptive summary of key information from all studies included in the review that will be presented in the form of a table. Additionally, numerical information regarding assessment tool usage will be presented through tables. A narrative study will be included to discuss the findings regarding barriers to using assessment tools and other findings that are more qualitative in nature. All results will also be described through the narrative summary.

### Dissemination

The findings from this scoping review will be published in an open-source, peer-reviewed journal. The research team also aims to disseminate the findings at national and international conferences.

### Study status

This scoping review is currently on Stage 4 of the JBI methodology for scoping reviews; ‘searching for the evidence’. The databases and search engines outlined in this protocol are being searched with the search strategy that can be seen in Table 2.

## Discussion

This protocol outlines the structure of a scoping review that will identify how validated assessment tools are used by nurses in general acute care settings to assess for delirium, and the barriers and enablers for said tools’ use. The scoping review will do so by investigating what validated assessment tools are most commonly used to assess for delirium, if there is any evidence of widespread usage of said tools, and what prevents or enables nurses’ using the tools. Should the scoping review differ from this protocol in any way, the ways in which and the reasons for will be acknowledged in the scoping review. The scoping review also aims to identify research gaps that can guide future researchers in the area of delirium.

## Ethics and consent

Ethical approval and consent are not required.

## Data Availability

No data are associated with this article.
