# Supplementary material for: Nurses’ usage of validated tools to assess for delirium in general acute care settings: A scoping review protocol
Source: HRB Open Res. 2025 Feb 13;8:33. [Version 1] doi: 10.12688/hrbopenres.14081.1 (PMC12103707; doi:10.12688/hrbopenres.14081.1)
Supplement: Peer review history

# Reviewer report: Reviewer response for version 1

**DOI:** 10.21956/hrbopenres.15468.r47013

**Published:** 2025-05-29

**Authors:** Tasia York

**Affiliations:** 1 Vanderbilt University, Nashville, USA

In this scoping review, the authors clearly describe their objectives to identify how validated assessment tools are used by nurses in general acute care settings to assess for delirium and to identify the barriers and enablers for the use of these tools. The authors note the rationale that the data shows the diagnosis of delirium is more accurate when utilizing a validated screening tool rather than personal judgement, and because delirium is associated with significant morbidity and mortality, improving our understanding of how nurses are currently screening for delirium will be beneficial in guiding future screening interventions.

 The study design applies the Joanna Briggs Institute (JBI) methodology to develop a structured protocol to answer their research questions and PRISMA-ScR to guide protocol development and reporting. The authors’ decision to include all study methodologies as well as grey literature and to utilize broad inclusion criteria will be beneficial in capturing a comprehensive summary of the available literature. They have provided precise details of their search strategy, study selection, data extraction and data analysis and presentation, and registered the scoping review protocol on Open Science Framework, thereby promoting transparency, increasing opportunities for replication by others, and reducing potential for publication bias.

 The design of this scoping review should highlight gaps in the literature and identify areas for future research.

Is the study design appropriate for the research question?

Yes

Is the rationale for, and objectives of, the study clearly described?

Yes

Are sufficient details of the methods provided to allow replication by others?

Yes

Are the datasets clearly presented in a useable and accessible format?

Not applicable

Reviewer Expertise:

child and adolescent psychiatry, consultation-liaison psychiatry, catatonia, delirium

I confirm that I have read this submission and believe that I have an appropriate level of expertise to confirm that it is of an acceptable scientific standard.

# Reviewer report: Reviewer response for version 1

**DOI:** 10.21956/hrbopenres.15468.r47005

**Published:** 2025-05-24

**Authors:** Tanya Mailhot

**Affiliations:** 1 Montreal Heart Institute, Montreal, Canada

The protocol titled *“Nurses’ usage of validated tools to assess for delirium in general acute care settings: A scoping review protocol”* outlines a well-conceived plan to synthesize current evidence on how nurses in general acute care settings use validated tools to assess for delirium, and to explore the barriers and enablers to such use. This review addresses a critical gap in the literature given the high prevalence and adverse outcomes of undetected delirium, and the fact that nurses are ideally positioned to conduct early assessments. The rationale for a scoping review is sound: the topic is broad, under-researched, and heterogeneous, and the goal is to map evidence rather than appraise interventions.

 The title clearly identifies the manuscript as a scoping review. The abstract is appropriately structured and includes the required elements. It is concise, informative, and aligned with the PRISMA-ScR recommendation for transparency.

 The introduction provides a detailed and well-referenced rationale, explaining the significance of delirium as a clinical problem and the current underutilization of validated assessment tools by nurses. The rationale for using a scoping review methodology is clearly stated and appropriate. The research objectives are explicitly formulated in relation to the population (registered nurses), the concept (validated delirium assessment tools), and the context (adult wards in general acute care hospitals), meeting PRISMA-ScR standards.

 Protocol registration is indicated and appropriately documented through a DOI on the Open Science Framework, ensuring transparency. The eligibility criteria are well defined and justified, including the time frame (2000–2025), language (English), population, and setting. These criteria are consistent with the review’s scope and research questions.

 The information sources are comprehensive and include CINAHL, PubMed, Scopus, Web of Science, and grey literature via Google Scholar and BASE. However, the protocol does not indicate the exact date of the most recent or planned search, which is a requirement of the PRISMA-ScR checklist. This information should be added to enhance reproducibility.

 A sample search strategy is provided in Table 2, demonstrating the use of Boolean operators and relevant keywords. This strategy is sufficient to meet the requirement of presenting a reproducible electronic search.

 The selection process for sources of evidence is described in detail, including dual independent screening, use of Covidence, piloting of the eligibility criteria, and a consensus process for resolving discrepancies. These procedures align with JBI recommendations and meet PRISMA-ScR expectations.

 The data charting process is articulated with a sample extraction tool (Table 3). Charting will be conducted independently by two reviewers, and the tool will be piloted and iteratively refined. While the data items are clearly described, the protocol could be strengthened by explicitly stating any assumptions or simplifications made during data charting.

 Consistent with scoping review methodology, the authors do not plan to conduct a critical appraisal of included studies. While this approach is appropriate, the rationale should be explicitly stated to comply fully with PRISMA-ScR item 12.

 The synthesis plan includes both tabular and narrative summaries, with the use of a PRISMA-ScR flow diagram to report the selection process. These methods are well aligned with the objectives and expected types of data.

 Although the results and discussion sections of the PRISMA-ScR are not applicable at the protocol stage, the authors describe in detail how they intend to present the findings, summarize key concepts and evidence gaps, and discuss implications for practice and policy.

 The funding source is clearly disclosed, and the authors confirm that the funder has had no role in the study design or conduct. This is appropriately reported in line with PRISMA-ScR item 22.

**Conclusion**

 This protocol demonstrates strong methodological rigor and relevance. It adheres well to the PRISMA-ScR checklist, with the following minor areas requiring attention:

1. Include the date of the most recent or planned database search.
2. Explicitly state that a critical appraisal of sources will not be conducted and provide justification.
3. Clarify any assumptions or simplifications in the data charting process.

These refinements would enhance clarity and ensure full alignment with PRISMA-ScR recommendations. Overall, the protocol is well developed and will likely generate valuable insights to inform clinical practice, education, and policy regarding delirium detection by nurses in acute care.

Is the study design appropriate for the research question?

Yes

Is the rationale for, and objectives of, the study clearly described?

Yes

Are sufficient details of the methods provided to allow replication by others?

Yes

Are the datasets clearly presented in a useable and accessible format?

Not applicable

Reviewer Expertise:

delirium, nursing

I confirm that I have read this submission and believe that I have an appropriate level of expertise to confirm that it is of an acceptable scientific standard.

# Reviewer report: Reviewer response for version 1

**DOI:** 10.21956/hrbopenres.15468.r46609

**Published:** 2025-04-14

**Authors:** Peter Lawlor

**Affiliations:** 1 Student Federation of the University of Ottawa, Ottawa, Ontario, Canada

The authors have submitted a protocol for a scoping review to examine the use of delirium screening instruments by nurses in general acute care settings. The authors aim to synthesize evidence on the basis of answers to the following questions:

 1. Is there evidence of the widespread usage of validated delirium assessment tools in general acute care settings by nurses?

 2. What delirium assessment tools are most commonly used by nurses in general acute care settings?

 3. What are the barriers and enablers to using validated delirium assessment tools that nurses experience in general acute care settings?

                    The role of a scoping review as opposed to a systematic review is often debated in relation to literature reviews. Systematic reviews invariably aim to answer a very specific and focussed research question. Scoping reviews, on the other hand, are arguably more forgiving of heterogeneity in terms of academic setting status (Teaching Hospital versus more general or District type hospital), patient groups or setting (Critical Care versus Geriatrics versus Emergency Department), and level of nurse expertise or training (if reported). Consequently, the scoping review methodology in this study will generate a broad literature map, and is largely appropriate to answer the three questions. The only the caveat is that it will likely answer the questions in relation to published studies, not necessarily what happens in routine nursing practice in general acute care. If there are no survey data among the included studies, the authors might also want to conduct a survey themselves to answer their three questions in relation to routine nursing practice, apart from research studies. One of the recognized roles of scoping reviews is that they identify gaps in the literature, and in this instance, pending the results, the authors might identify the need for surveys and other primary studies, such as focus groups and other qualitative methods.

Is the study design appropriate for the research question?

Yes

Is the rationale for, and objectives of, the study clearly described?

Yes

Are sufficient details of the methods provided to allow replication by others?

Partly

Are the datasets clearly presented in a useable and accessible format?

Not applicable

Reviewer Expertise:

Delirium, cancer pain management, opioid toxicity and palliative care

I confirm that I have read this submission and believe that I have an appropriate level of expertise to confirm that it is of an acceptable scientific standard.
